# MRI image sequencing of calcified myocardial masses: liquefaction necrosis of mitral annular calcification (LNMAC)

**DOI:** 10.1186/1532-429X-13-S1-P353

**Published:** 2011-02-02

**Authors:** David A Collins, Melissa King-Strunk, Wojciech Mazur, Amy Tipton, Sanjay S Srivatsa

**Affiliations:** 1 The Christ Hospital, Cincinnati, OH, USA; 2 Cincinnati Childrens Hospital Medical Center, Cincinnati, OH, USA; 3 Marion Medical Center, Santa Maria, CA, USA

## Introduction

LNMAC presents as a cardiac mass with liquefied necrotic core and a thick inflammatory/fibrotic/calcified capsule peripherally.

## Purpose

To characterize the diagnostic MRI sequences most useful in diagnosis of calcified myocardial masses.

## Methods

CMRI imaging utilized a dedicated 8 cardiac coil system, the Siemens Avanto™ 1.5Tesla MR scanner and gadolinium contrast. HASTE sequence (myocardial morphology), inversion recovery, Turbo FLASH and phase sensitive inversion recovery and True FISP sequences were utilized.

## Results

The imaging characteristics are summarized in Table 1.

**Table 1:** MRI Imaging Characteristics of LNMAC

MRI SEQUENCE USED TO ASSESS LNMAC	INTERIOR CHARACTERISTIC OF MASS BY MRI	PERIPHERAL RING ENHANCEMENT
PHASE SENSITIVE/MAGNITUDE RECONSTRUCTION INVERSION RECOVERY (PSIR) WITH MYOCARDIAL NULLING	HYPOINTENSE‡ ‡signal intensity of central part of mass compared with surrounding myocardium	+++ (marked PERIPHERAL LATE GADOLINIUM ENHANCEMENT)
HASTE (T2 WEIGHTED SINGLE SHOT FSE)	HYPOINTENSE‡ WITH HETEROGENEOUS INTERIOR COMPONENTS	+++ (marked PERIPHERAL LATE GADOLINIUM ENHANCEMENT)
IR TURBOFLASH (ULTRA FAST GRADIENT LOW ANGLE SHOT T1 WEIGHTED)	HYPOINTENSE‡ WITH HETEROGENEOUS INTERIOR COMPONENTS	+++ (marked PERIPHERAL LATE GADOLINIUM ENHANCEMENT)
GRE (SPOILED GRADIENT ECHO)	HYPERINTENSE CENTER AND HYPOINTENSE RIM‡	NONE
TRUE FISP (STEADY STATE GRE MIXED T1 AND T2 WEIGHTING)	HYPOINTENSE‡	NONE
INVERSION RECOVERY TRUE FISP (SINGLE SHOT NON-BREATH HOLD TECHNIQUE	HYPOINTENSE‡	++
STIR (SHORT TAU IR OR TRIPLE INVERSION RECOVERY)	HYPOINTENSE‡	++
T2 TURBO SPIN (DARK BLOOD) ECHO (TSE)	HYPOINTENSE‡	++
T1 TURBO SPIN ECHO FAT SAT POST CONTRAST DARK BLOOD (TSE)	HYPOINTENSE‡ WITH HETEROGENEOUS INTERIOR COMPONENTS	NONE

## Conclusions

**Figure 1:**
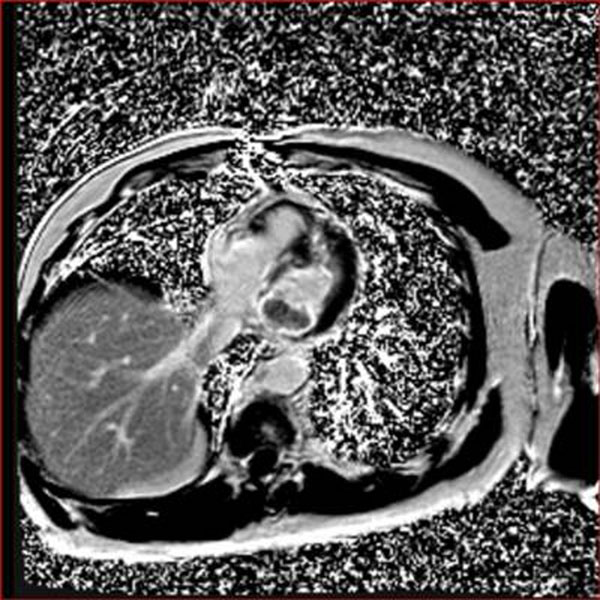
Horizontal long axis 4 chamber view: PSIR

**Figure 2:**
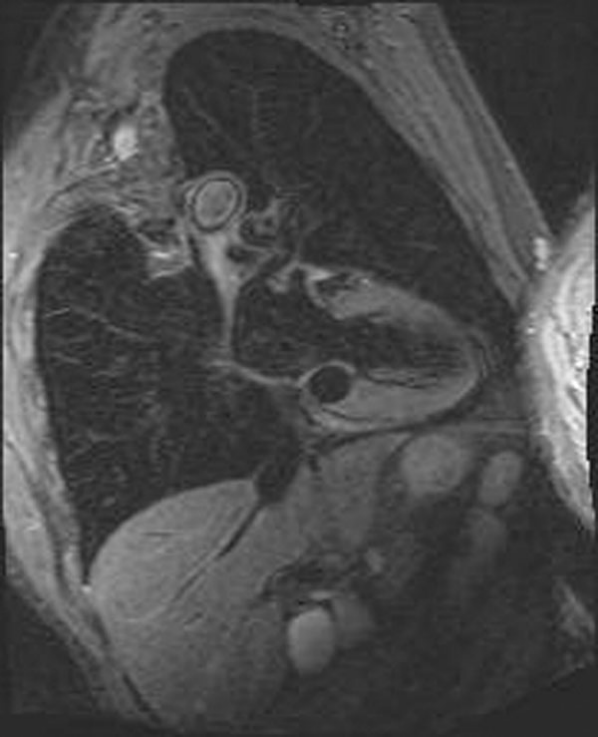
STIR imaging using a short inversion time to suppress fat

**Figure 3:**
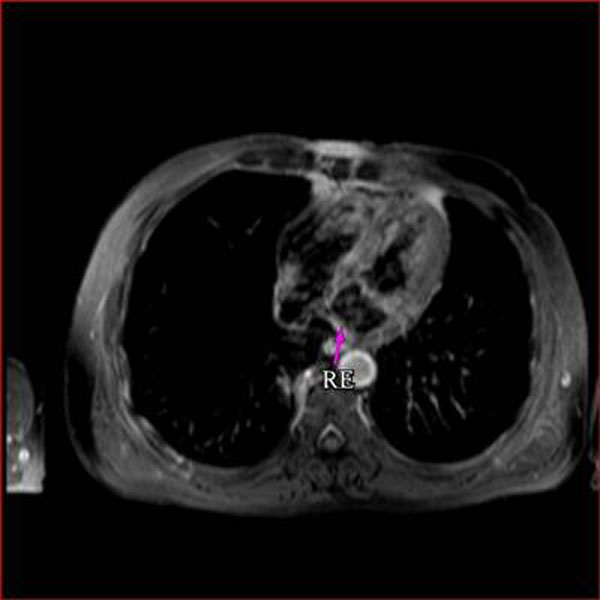
T1 weighted, post contrast, dark blood, fat saturated, turbospin echo

**Figure 4:**
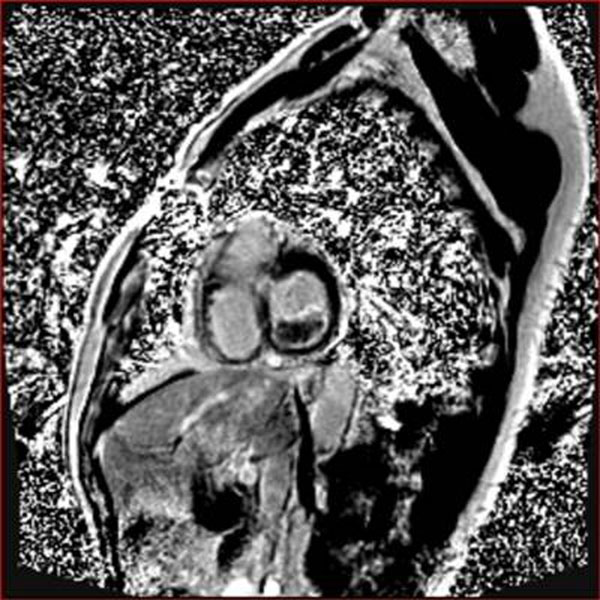
PSIR short axis cross-section at mitral valve annulus

**Figure 5:**
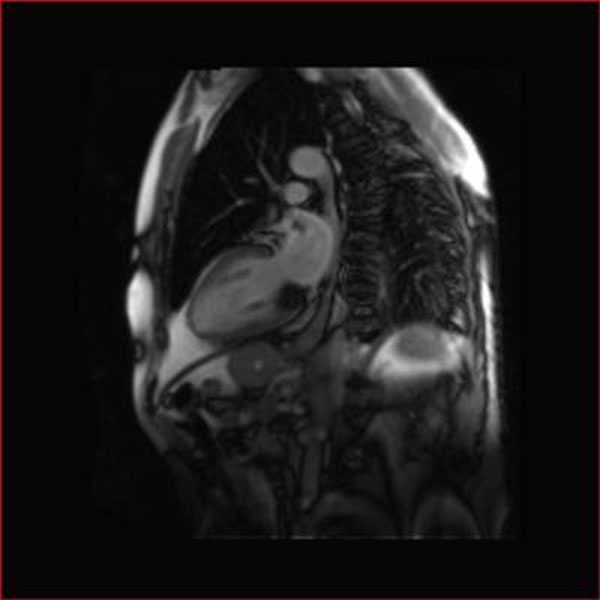
2 chamber T1 weighted image demonstrating hypointense lesion compared with surrounding myocardium.

**Figure 6:**
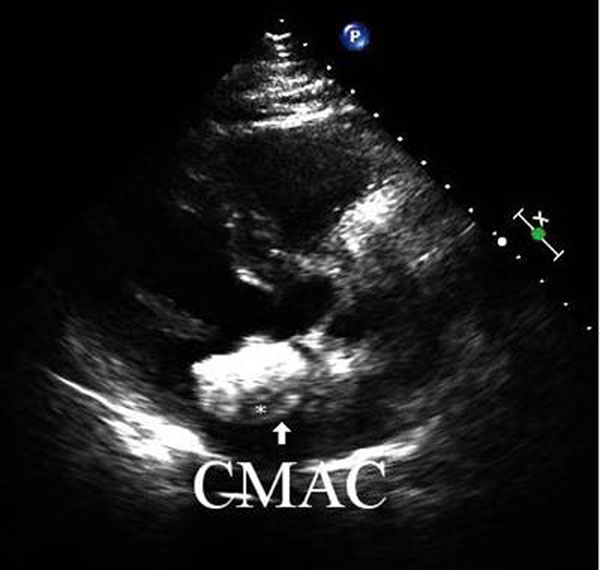
Transthoracic echocardiography two dimensional parasternal long axis

**Figure 7:**
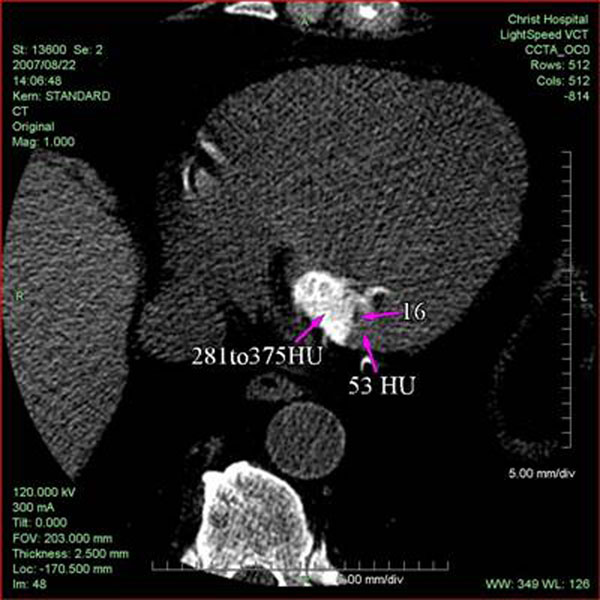
High resolution thin section CT axial view

LNMAC histopathology reveals a necrotic core (amorphous eosinophilic material) and surrounding inflammatory rim (macrophage and lymphocytic infiltration) with zonal calcification. By MRI this zone of cellular inflammation and edema manifests as the zone of peripheral hyperenhancement on the PSIR, HASTE, and IR TURBOFLASH sequences. Most benign or malignant cardiac tumors show high T2 weighted imaging intensity, and are myocardial isointense on T1 weighted imaging before contrast administration. In contrast, the hypointense central zone of liquefactive necrosis seen on T1 and T2 weighted sequences, and the surrounding bright ring like zone of hyperintensity seen with both T2 weighted HASTE and phase sensitive (PSIR)inversion recovery sequences, serves along with its typical location and calcified content to distinguish it from other cardiac tumors. Shortening the inversion time of the inversion recovery sequence (STIR), enhances sensitivity to certain types of pathology by making the effects of prolonged T1 and T2 on signal intensity additive and nulling fat signal. Both tumor and inflammatory pathologies may therefore be separated in terms of contrast, from fat and muscle as in this case. The T2-weighted edema imaging using a breath-hold single-shot sequence with half-Fourier imaging (HASTE) maps the ring like area of pronounced inflammation and associated edema, surrounding the central area of liquefactive necrosis, which is comparatively less intense compared with the outer ring zone. Likewise, a breath hold inversion recovery segmented turbo fast low-angle (IR turboFLASH) sequence for T1-weighted postcontrast imaging, or T2-weighted short TI inversion-recovery precontrast imaging (STIR) both yield a well defined ring like area of signal enhancement around a hypointense core. These MRI sequence characteristics are diagnostic of LNMAC. Figures 1, 2, 3, 4, 5, 6, 7.

